# Adalimumab induced psoriasis in Crohn’s disease and treatment with ustekinumab: case report and special histopathological findings

**DOI:** 10.3389/fmed.2024.1507524

**Published:** 2025-01-24

**Authors:** Fang Qiu, Weiquan Chen, Ying Zhou

**Affiliations:** Institute of Dermatology and Venereal Diseases, Affiliated Hospital of Guangdong Medical University, Zhanjiang, China

**Keywords:** TNF-α inhibitor induced psoriasis, adalimumab, Crohn’s disease, ustekinumab, case report

## Abstract

This case report presents an instance of Tumor Necrosis Factor-*α* Inhibitor-induced psoriasis (TNFiIP), also known as paradoxical psoriasis, in a 30-year-old male with fistulizing Crohn’s disease. The patient developed extensive erythematous and scaly lesions on the palms, lower limbs, ankles, and soles after 4 months of adalimumab monotherapy. Histopathological analysis revealed a pattern of psoriasiform dermatitis with notable dermal neutrophil and eosinophil infiltration, distinguishing TNFiIP from idiopathic psoriasis. The patient’s condition significantly improved following the transition from adalimumab to ustekinumab, which highlights the importance of alternative therapeutic strategies for patients who exhibit paradoxical reactions to TNF-*α* inhibitors.

## 1 Introduction

Crohn’s disease (CD) is a chronic inflammatory bowel disease with symptoms such as cramping abdominal pain, chronic diarrhea, fever, anemia-related fatigue, and weight loss. These symptoms can persist for days or weeks, often recurring and potentially leading to serious complications like bowel obstruction abdominal abscesses, fistulas, anal fissures, and colon cancer (1). Adalimumab, a fully human monoclonal antibody targeting tumor necrosis factor (TNF)-*α*, is one of the widely used TNF-*α* inhibitors in the treatment of CD and psoriasis (2). This case report presents an instance of the paradoxical induction of psoriasis by TNF-α inhibitors (TNFis) and discussed the clinical, pathological features and therapeutic considerations for TNFis induced psoriasis (TNFiIP), including the assessment of the necessity of discontinuing TNFi, topical corticosteroid therapy, and exploration of alternative biologics such as IL⁃12/IL⁃23 inhibitors. The successful transition to ustekinumab after the development of TNFiIP highlights the importance of alternative therapeutic strategies for patients who exhibit paradoxical reactions to TNF-*α* inhibitors. The shift from broad immunosuppression (via TNF-α inhibitors) to more specific immune modulation with ustekinumab may help avoid further exacerbations of paradoxical psoriasis while effectively controlling the underlying condition (3).

## 2 Case report

**Figure 1:**
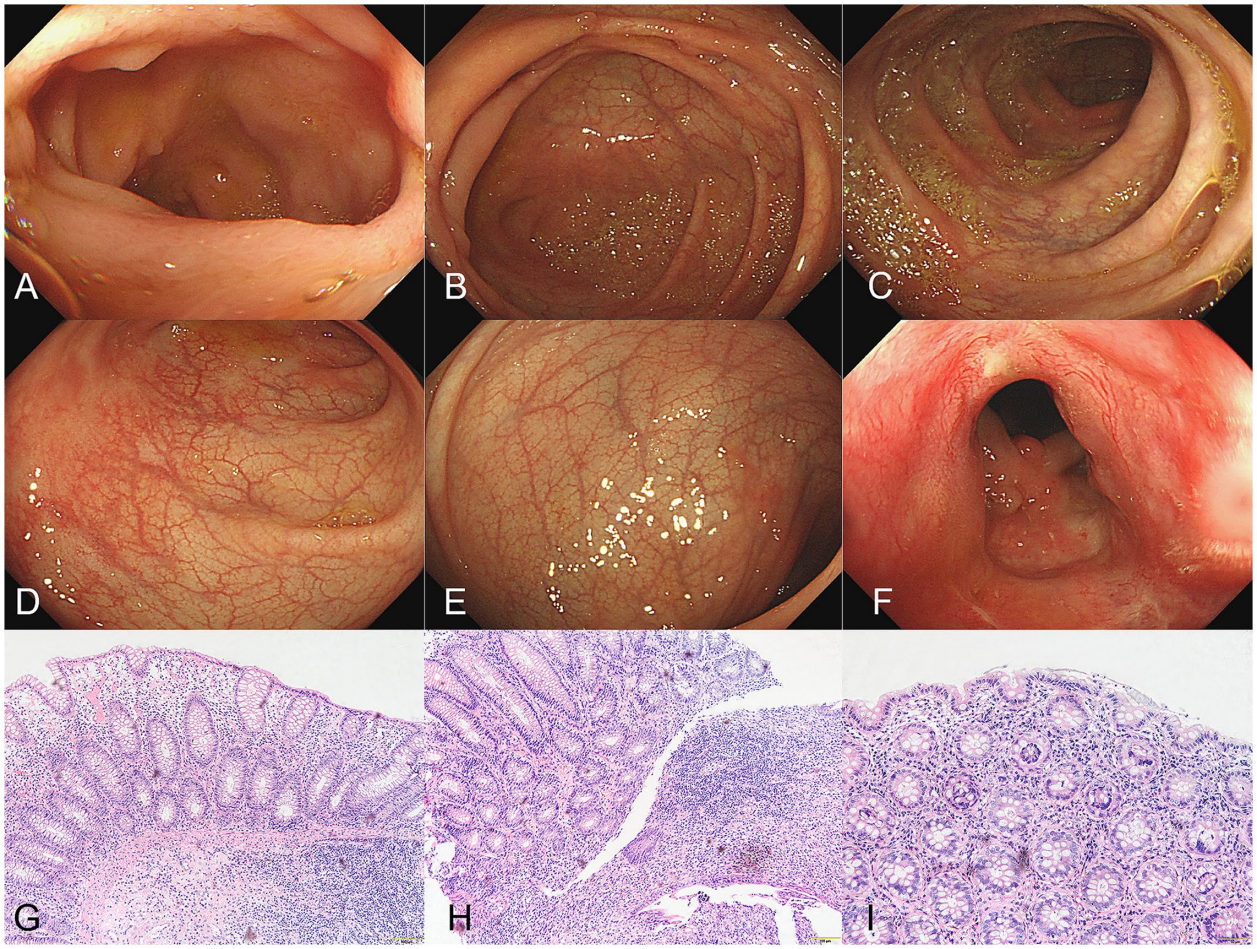
**(A–F)** The report of the colonoscope describes scattered ulcers, scars, and polypoid hyperplasia observed in the mucosa. **(G–I)** Microscopic images of the patient’s colon tissue section revealed colonic mucosa with crypt abscesses, crypt architectural distortion, and expansion of the lamina propria with mixed inflammatory cell infiltration (hematoxylin&eosin, **G**, magnification ×100, **H**,**I**, magnification × 200).

**Figure 2:**
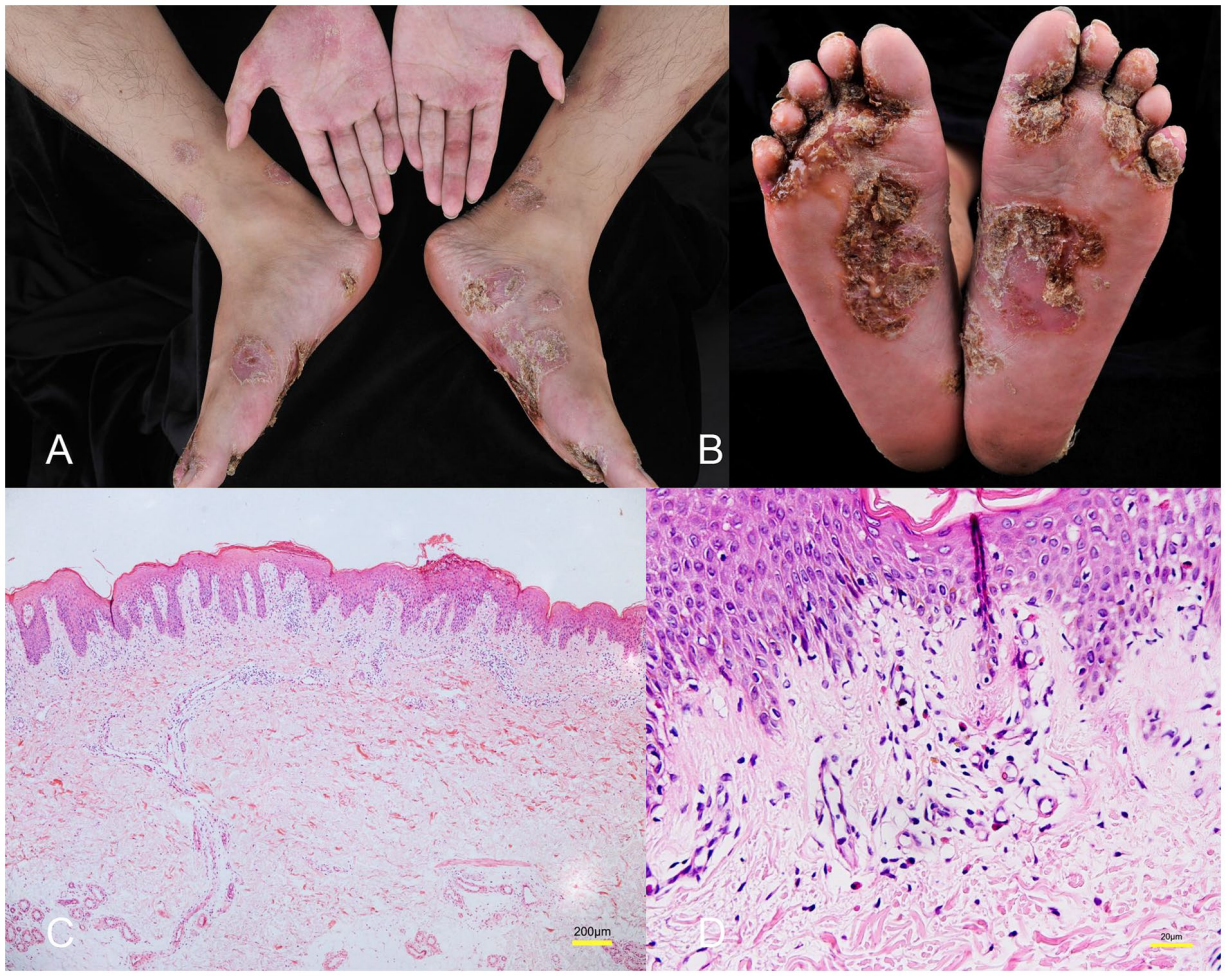
**(A,B)** Multiple scaly erythematous eruptions and pustules on the ankles, lower legs, knees, elbows, palms, and soles. **(C)** Histology showing epidermal psoriasiform hyperplasia, Kogoj’s micro-abscesses and spongiotic vesicle in the epidermis (hematoxylin&eosin, magnification ×40). **(D)** Eosinophils observed in the dermis (hematoxylin&eosin, magnification ×400).

**Figure 3:**
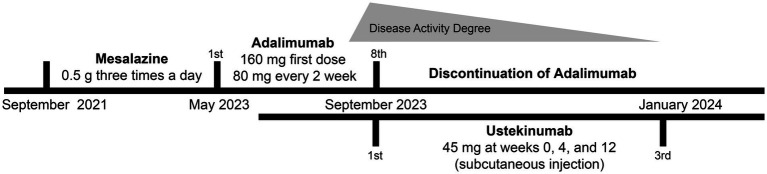
Timeline of the patient’s diagnosis with the relevant data about the treatment and follow-up.

A male patient in his early 30s with a 2-year history of fistulizing Crohn’s disease (CD) was unresponsive to conventional treatment, which included mesalazine 0.5 g three times daily for 10 months. The patient has no history of smoking or alcohol consumption, and no comorbidities. Investigation into his family history revealed that none of his close relatives have been diagnosed with CD, psoriasis, or exhibit similar symptoms. The report of the colonoscope describes scattered ulcers, scars, and polypoid hyperplasia observed in the mucosa (Figures 1A–F). Microscopic images of the patient’s colon tissue section reveal crypt distortion and abscesses (Figures 1G–I). He was started on adalimumab monotherapy. The dosing regimen included an initial subcutaneous injection of 160 mg, followed by 80 mg subcutaneous injections maintained every 2 weeks thereafter. Four months after initiating adalimumab treatment, the patient developed multiple scaly erythematous eruptions and pustules on the ankles, lower legs, knees, elbows, palms, and soles (Figures 2A,B). A skin biopsy of the plaque was performed revealing epidermal psoriasiform hyperplasia, Kogoj’s micro-abscesses and spongiotic intraepidermal vesicle, and neutrophils in the vesicle, eosinophils observed in the dermis (Figures 2C,D). Adalimumab was discontinued, and the patient was transitioned to ustekinumab for CD management, administered as a 45 mg subcutaneous injection at weeks 0, 4, and 12, alongside fluticasone cream for psoriasis treatment. The comprehensive medical history, clinical examination, and treatment response supported the diagnosis of TNF-*α* inhibitor-induced psoriasis. Four months later, the patient’s psoriatic lesions resolved, and his CD entered remission (Figure 3).

## 3 Discussion

TNF-α inhibitors, which are effective in treating psoriasis, paradoxically induce psoriasis themselves, termed TNFiIP, also named paradoxical psoriasis. Dendritic cells (DCs) producing TNF and IL-23 activate CD4^+^ and CD8^+^T-cells, leading to their migration into the epidermis. Upon recognizing autoantigens, Tcells produce Th17 cytokines, including IL-17A, IL-17F, and IL-22, fostering a classic psoriatic phenotype by inducing keratinocyte hyperproliferation (4).

Classic psoriasis is considered a T-cell-mediated autoimmune disease driven by TNF, while TNFiIP is induced by TNF deficiency. Immature plasmacytoid DCs (pDCs) contribute to early-stage inflammation in psoriasis by producing Type I IFNs, which stimulate autoreactive T cell amplification, notably CD8^+^T cells (4). These self-reactive T cells migrate to the epidermis, recognizing autoantigens of keratinocytes, subsequently inducing hyperproliferation of keratinocytes (5). TNF induces pDC maturation, thereby upregulating co-stimulatory molecules and diminishing their ability to produce interferon. Conversely, TNF blocking reduces pDC maturation and prolongs IFN release (6). Elevated pDCs and type I IFN levels are observed in TNFiIP skin lesions; the accumulation of pDCs also correlates with the expression levels of type I IFNs (7). However, the precise pathogenic mechanisms triggering pDC activation in TNFiIP remain incompletely understood, necessitating further research. Among TNFiIP, palmoplantar pustulosis represents a distinct subset. The mixed Th2/Th17 component is characteristic of classical palmoplantar pustulosis. Therefore, TNF-*α* blockade, by inhibiting the IL-17 axis, may result in an overexpression of Th2 cytokines, potentially triggering palmoplantar pustulosis in susceptible individuals (8).

Pathological features such as epidermis thinning above the dermal papillae and the neutrophil presence in the stratum corneum are considered more common in classic psoriasis. Conversely, >3 eosinophils in the dermis, spongiosis and pustule are commonly observed in TNFiIP, which is similar to atopic eczema and might serve as diagnostic clues (9). Several studies have consistently documented eosinophil presence in TNFiIP biopsy tissues, aligning with our case (10). The underlying pathogenic mechanisms also support eosinophil involvement. Akin to TNF-*α*, eosinophils can inhibit pDC production of type I interferon. With TNF is use diminishing TNF-α’s inhibitory effect, there’s a localized increase in pDCs and type I interferon, leading to compensatory eosinophil recruitment and accumulation in the dermis (11). However, further studies to validate these observations in larger cohorts are still needed.

For treatment, it’s recommended to first assess the necessity of discontinuing or switching TNFi, topical corticosteroid therapy may be considered, possibly combined with phototherapy or systemic treatment. In refractory cases, IL-12/IL-23 inhibitor demonstrates promising efficacy against TNFiIP (12). In this case, the patient experienced not only rapid and sustained relief from skin lesions but also CD following ustekinumab treatment. Of note, TNFiIP has also been reported in patients undergoing ustekinumab treatment (13).

As a single case report, the findings cannot be generalized to broader patient populations. Additionally, the lack of a control group and statistical analysis limits the ability to establish causality or draw definitive conclusions. Further large-scale studies are needed to confirm the efficacy and safety of ustekinumab in managing paradoxical psoriasis and Crohn’s disease. Due to the insufficient investigation of the genetic susceptibility of TNFiIP patients, future identification may involve genetic variations driving pDC, type I IFN signaling transduction, and T-cell autoimmune activation.

## 4 Conclusion

If scaly erythematous macules and pustules develop on the extremities or palms during anti-TNF-*α* therapy, TNFiIP should be considered as a potential diagnosis. Histopathologically, TNFiIP exhibits features of idiopathic psoriasis along with epidermal spongiosis, pustules, and infiltration of dermal eosinophils. Compared to idiopathic psoriasis, TNFiIP may show improvement upon discontinuation or switching to alternative biologics, with fewer relapses.

## Data Availability

The original contributions presented in the study are included in the article/supplementary material, further inquiries can be directed to the corresponding author/s.
